# Single-Fraction Stereotactic Body Radiotherapy for Early-Stage Lung Adenocarcinoma Within Atelectasis

**DOI:** 10.7759/cureus.107819

**Published:** 2026-04-27

**Authors:** Joseph Daniels, Benjamin Mou

**Affiliations:** 1 Radiation Oncology, BC Cancer Kelowna, Kelowna, CAN

**Keywords:** atelectasis, collapsed lung, consolidation, early-stage lung cancer, non-small cell lung cancer, sabr, sbrt, single fraction, stereotactic ablative radiotherapy, stereotactic body radiotherapy

## Abstract

Stereotactic body radiotherapy (SBRT) is an established non-invasive treatment for early-stage non-small cell lung cancer (NSCLC) in patients who are medically inoperable or who decline surgery. However, delivering SBRT to tumors within atelectasis presents unique challenges due to anatomical distortion, impaired tumor visualization, and uncertainties in target motion and localization. A 61-year-old woman with severe chronic obstructive pulmonary disease on long-term home oxygen therapy was diagnosed with biopsy-confirmed right upper lobe lung adenocarcinoma located within an area of atelectasis. She was medically inoperable due to critically reduced pulmonary reserve. Staging positron emission tomography-computed tomography (PET-CT) revealed a focal hypermetabolic right upper lobe lesion with no evidence of nodal or distant metastases. The patient received single-fraction SBRT (SF-SBRT) to 34 Gy using a 1 cm isotropic planning target volume margin to account for increased planning and treatment-related uncertainties. Target delineation was guided by PET-CT to distinguish metabolically active tumor from surrounding atelectasis, and image guidance was performed using cone-beam CT matching to surrogate anatomic landmarks due to limited direct tumor visualization. At 6.1 months after SBRT, the patient demonstrated significant metabolic response on PET-CT but developed rapidly progressive brain metastases and died 6.6 months after SBRT. This case demonstrates the feasibility of SF-SBRT for early-stage NSCLC within atelectasis. While short-term metabolic response was achieved, long-term local control remains unclear and further research into the management of lung tumors within atelectasis is warranted.

## Introduction

Stereotactic body radiotherapy (SBRT) is an established non-invasive treatment for early-stage non-small cell lung cancer (NSCLC) in patients who are medically inoperable or who decline surgery. This approach is supported by randomized clinical trials, large institutional series, and population-based studies showing excellent local control rates of about 90% and favorable toxicity profiles, including elderly and high surgical risk cohorts [1-4]. Despite strong evidence, significant gaps remain in the literature regarding lung SBRT in anatomically challenging situations, such as lung tumors embedded within atelectasis.

Tumors in atelectasis pose challenges due to impaired visualization, distorted anatomy, altered tissue density, uncertainties in target motion, and the possibility that re-expansion may shift the target [5]. Atelectasis complicates tumor delineation and dosimetric accuracy, increasing uncertainty in both target definition and organ-at-risk sparing. Modern treatment planning incorporates advanced imaging, such as 4‑dimensional computed tomography (4D‑CT) and positron emission tomography-computed tomography (PET-CT), to accurately assess motion and guide tumor delineation. This approach improves target delineation and reduces the risk of geographic miss; however, supporting evidence in the setting of atelectasis is limited. Patients with early-stage NSCLC within atelectasis are not well described in the literature, and evidence guiding optimal target margins, motion management strategies, and image-guided target localization in this setting remains scarce.

This case report describes the use of single-fraction SBRT (SF-SBRT) in a medically inoperable patient with early-stage NSCLC located within atelectasis, highlighting key technical considerations in imaging, target delineation, and treatment delivery.

## Case presentation

A 61-year-old woman with severe chronic obstructive pulmonary disease (COPD) on long-term home oxygen therapy presented with hemoptysis, cough, and dyspnea. She denied fever, chest pain, or significant weight loss. Her medical history was notable for chronic hypoxemia, hypertension, and recurrent COPD exacerbations treated with systemic corticosteroids. She had no history of prior thoracic surgery or radiotherapy. She was an ex-smoker with a 45-pack-year history and stopped smoking five years prior to presentation. Vital signs were stable, with oxygen saturation of 93% on supplemental oxygen at 3 L/min. Pulmonary examination revealed decreased breath sounds and wheezing bilaterally. Her Eastern Cooperative Oncology Group performance status was 2 [6].

**Figure 1:**
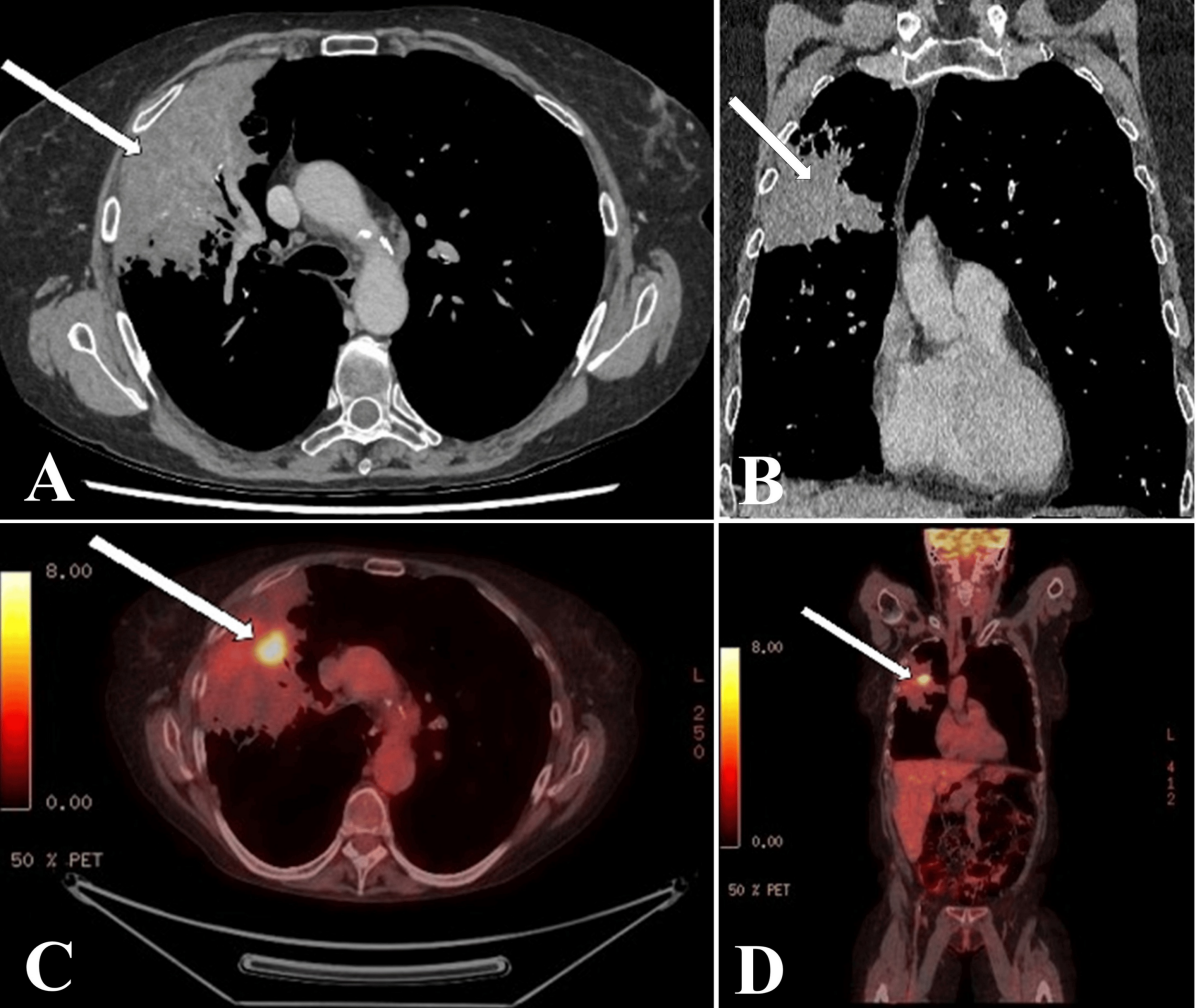
Diagnostic imaging (A) Axial and (B) coronal views of the initial diagnostic chest computed tomography scan. (C) Axial and (D) coronal views of the staging positron emission tomography-computed tomography scan. The white arrows indicate the hypermetabolic primary tumor with surrounding non-avid atelectasis.

Initial chest radiography demonstrated consolidation within the right upper lobe, thought to represent pneumonia or possible alveolar hemorrhage given the clinical history. A trial of antibiotics was initiated, and serial chest radiographs were performed. However, there was no improvement in the patient’s symptoms or radiographic findings. Subsequently, a diagnostic chest CT demonstrated persistent right upper lobe consolidation in the setting of severe emphysema, extending to the right middle lobe (Figure 1A, 1B). Bronchoscopy showed no endobronchial disease, while cytological examination of bronchial brushings and washings demonstrated atypical cells without evidence of malignancy. Pulmonary function testing demonstrated a severely reduced forced expiratory volume in 1 second (FEV1) of 0.35 L (16% predicted). Staging PET-CT scan was performed in accordance with standard institutional protocols and demonstrated extensive right upper and middle lobe consolidation (approximately 17 cm) with minimal metabolic activity. Within the consolidation in the right upper lobe, there was a 2.4 cm focus of high-grade ¹⁸F-fluorodeoxyglucose (FDG) uptake (maximum standard uptake value [SUV] 10.4) (Figure 1C, 1D). There was no evidence of FDG-avid lymphadenopathy or distant metastasis.

**Figure 2:**
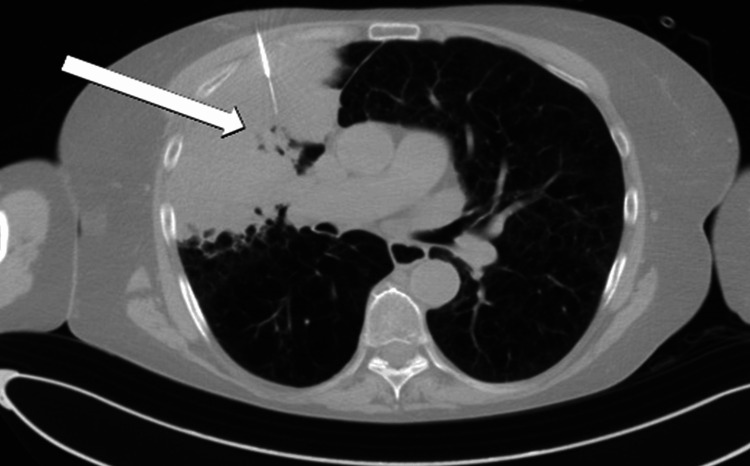
Computed tomography-guided percutaneous biopsy of the right upper lobe consolidation The white arrow indicates the part of the tumor within atelectasis that was biopsied confirming pulmonary adenocarcinoma.

A CT-guided biopsy (Figure 2) confirmed lung adenocarcinoma, though the sample was not adequate for molecular analysis. Head CT scan was negative for metastases. She was staged as clinical T1c N0 M0 right upper lobe lung adenocarcinoma and discussed at a multidisciplinary tumor board where the consensus recommendation was to treat with lung SBRT.

**Figure 3:**
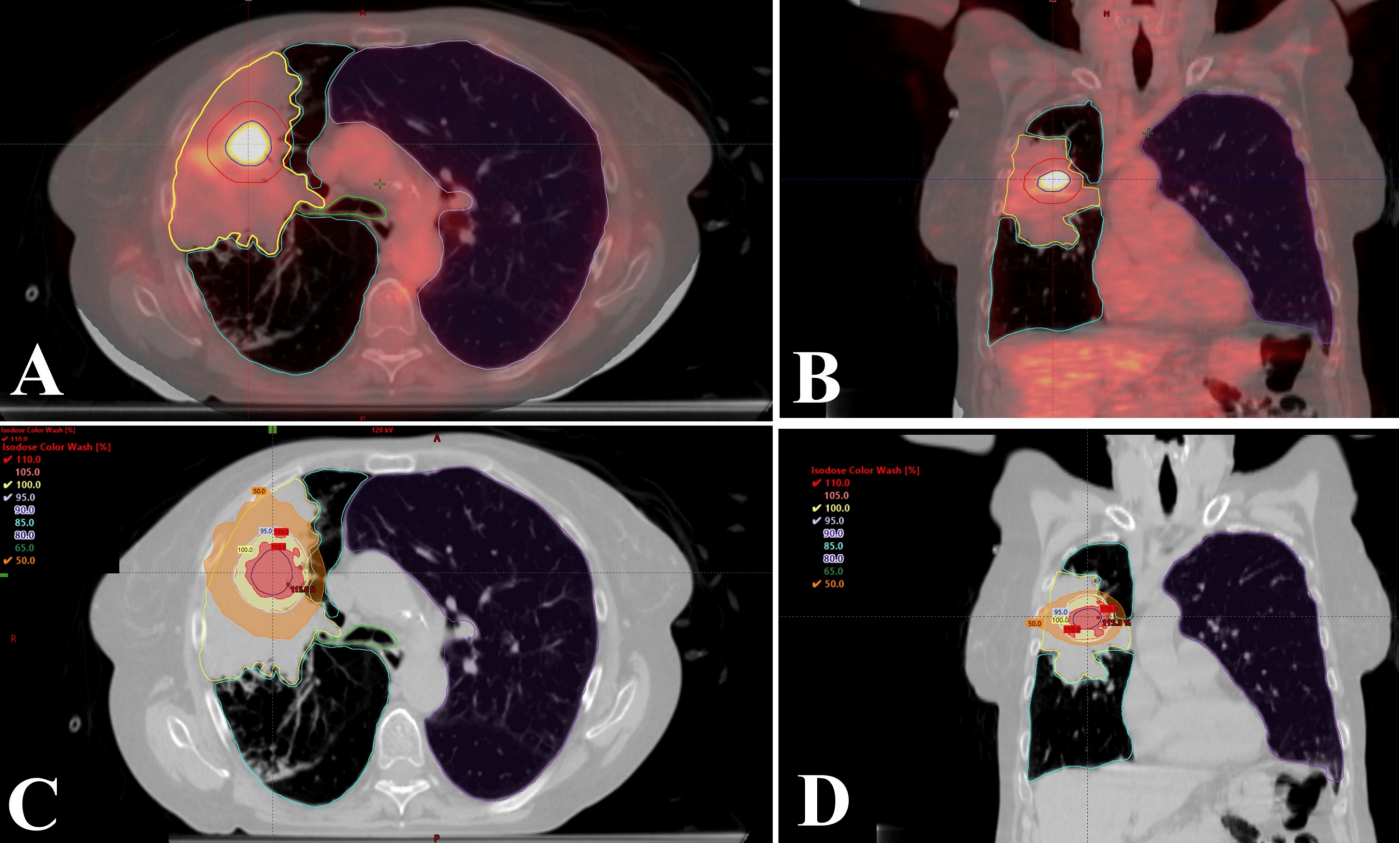
Treatment planning (A) Axial and coronal (B) views of the delineated gross tumor volume (blue) and planning target volume (red) on the fused positron emission tomography-computed tomography scans. (C) Axial and (D) coronal views of the radiation treatment plan showing the dose coverage of the gross tumor volume and planning target volume. Also shown are the region of atelectasis (yellow), distal bronchus (green), ipsilateral (cyan) and contralateral lungs (purple).

The patient was simulated with a 4D-CT scan and abdominal compression per institutional policy. Direct tumor visualization on the planning CT for gross tumor volume (GTV) delineation was significantly impaired by the surrounding atelectasis, making contouring challenging. PET-CT image fusion was performed by standard rigid PET-CT registration, which improved tumor delineation (Figure 3A, 3B). Assessment of the 4D-CT scan demonstrated minimal motion of the atelectasis. The GTV was contoured as the area of focal FDG uptake excluding adjacent non-avid atelectatic lung. An internal target volume (ITV) was not contoured due to insignificant motion and challenges with accurate delineation within the atelectasis. The planning target volume (PTV) was generated by an isotropic 1 cm expansion from the GTV. This generous margin was selected to account for increased uncertainties in tumor delineation, localization within surrounding atelectasis, and respiratory motion. The volumes of the GTV and PTV were 6.0 cc and 42.8 cc, respectively. The patient was treated with SBRT to a dose of 34 Gy in one fraction using 10 MV flattening filter-free photons delivered on an Edge linear accelerator (Varian Medical Systems, Palo Alto, CA, USA) with a volumetric modulated arc therapy technique. Greater than 95% of the PTV received 100% of the prescribed dose (Figure 3C, 3D).

Normal tissue limits adhered to institutional dose constraints based on the SF arm of the Radiation Therapy Oncology Group 0915 randomized trial and were prioritized over target volume coverage [4,7]. A summary of dosimetric planning goals and achieved metrics is listed in Table 1. Pre-treatment cone-beam CT (CBCT) was used for image guidance, matching to surrogate adjacent anatomic landmarks, specifically the atelectasis itself and distal segments of the right upper lobe bronchi adjacent to the GTV.

**Table 1:** Dosimetric parameters GTV: Gross tumour volume,  PTV: Planning target volume, Vx Gy: Volume receiving at least x Gy, Dmax: Maximum dose to 0.035 cc, D1500 cc: minimum dose to the highest-irradiated 1500 cc

Structure	Planning Objective	Result
GTV	V34 Gy > 100.0%	100.00%
	Dmax > 111.0%	115.98%
	Dmax ≤ 150.0%	115.98%
PTV	V34 Gy > 95.0%	96.48%
	V30.6 Gy > 99.0%	100.00%
	Dmax ≤ 150.0%	116.00%
	V99.5% > 50.0%	97.20%
Brachial plexus	Dmax ≤ 14 Gy	0.13 Gy
Chest wall	V18 Gy ≤ 30 cc	3.09 cc
	Dmax ≤ 26 Gy	21.91 Gy
Spinal canal	Dmax ≤ 12.4 Gy	3.44 Gy
Great vessels	Dmax ≤ 26 Gy	10.24 Gy
Heart	Dmax ≤ 18 Gy	14.26 Gy
	V15 Gy ≤ 15 cc	0.00 cc
Lungs	V11 Gy ≤ 10.0%	0.28%
	Dmean ≤ 4 Gy	0.82 Gy
	D1500 cc ≤ 7 Gy	0.84 Gy
Proximal bronchial tree	Dmax ≤ 18 Gy	11.15 Gy
Proximal trachea	Dmax ≤ 18 Gy	5.29 Gy
Skin	Dmax ≤ 19 Gy	10.00 Gy
	V18 Gy ≤ 10.0 cc	0.00 cc

**Figure 4:**
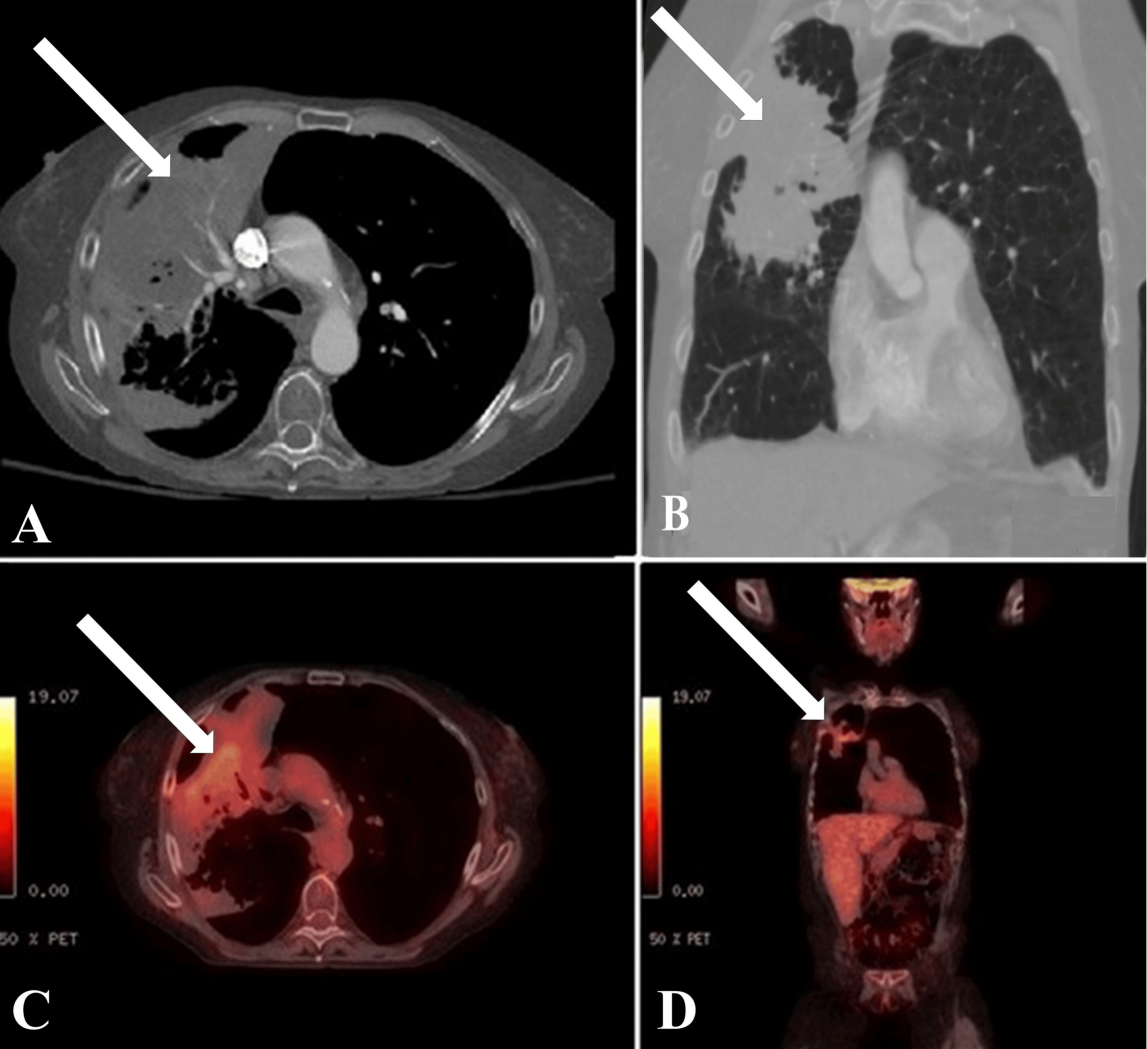
Follow-up imaging post treatment (A) Axial and (B) coronal views of the follow-up chest computed tomography scan. (C) Axial and (D) coronal views of the follow-up positron emission tomography-computed tomography scan.

The patient completed SF-SBRT as intended without acute complications and remained clinically stable without new respiratory symptoms. The patient subsequently experienced neurological decline and cranial imaging at 5.6 months after SBRT demonstrated a 2.3 cm solitary brain metastasis in the left posterior frontal lobe. Re-staging PET-CT at 6.1 months after SBRT demonstrated metabolic resolution of the focal lesion within the area of consolidation in the right upper lobe (SUVmax 5.1) consistent with interval metabolic response, and no progressive local or distant FDG-avid lesions were identified (Figure 4).

Craniotomy with tumor resection was initially considered but not pursued due to her extremely high operative risk and poor likelihood of post-operative recovery. Treatment with stereotactic radiosurgery was planned but could not be delivered because the patient was hospitalized with delirium, mania, and coronavirus disease 2019 (COVID-19) infection requiring increased oxygen requirements. In light of her rapidly declining performance status, the patient chose to transition to palliative care and died 6.6 months after SBRT. 

## Discussion

This case demonstrates the feasibility of SF-SBRT in peripheral early‑stage NSCLC within atelectasis. Atelectasis presents unique challenges for radiotherapy, as malignant lesions embedded within non‑aerated lung are difficult to visualize. The loss of normal aeration obscures tumor boundaries, distorts anatomic landmarks, and complicates respiratory motion assessment. Tumor‑related atelectasis has been identified as a negative prognostic factor for overall survival in patients with NSCLC treated with radiotherapy, reflecting more aggressive disease biology rather than the efficacy of local therapy [8]. Conventional CT imaging often cannot reliably distinguish tumor from atelectasis, which can lead to over‑contouring if the entire area of atelectasis is included in the GTV [9]. PET-CT enhances target definition by incorporating metabolic information to distinguish viable tumor from surrounding non-malignant tissue, thereby reducing geographic miss and interobserver variability [10,11]. PET-CT co-registration was critical for accurate GTV delineation within extensive consolidation, enabling clear separation of metabolically active tumor from adjacent atelectasis and improving confidence in target definition. This approach has been demonstrated to optimize radiotherapy planning in patients with stage III NSCLC and tumor-related atelectasis undergoing three-dimensional conformal radiotherapy [9,12] and patients with central lung tumors treated with SBRT [13].

The optimal approach to contouring collapsed lung remains uncertain as there are discrepancies from prospective cooperative group clinical trials [4,14]. While excluding non-functional, atelectatic lung from the GTV can reduce overall target volume and normal lung dose [12], there is a theoretical risk that inter-fraction re-expansion of the lung could lead to unintended irradiation of aerated lung tissue, particularly in multi-fraction regimens [5,15]. The decision to include or exclude atelectatic lung from SBRT planning is individualized and guided by PET-CT metabolic information, tumor location, and anticipated potential lung re-expansion. This highlights an area of clinical uncertainty and underscores the need for further research to standardize target delineation in tumors embedded within atelectasis. The choice of a SF-SBRT regimen limits risks associated with inter-fraction anatomic changes due to tumor response or airway reopening, which could otherwise result in target displacement and dosimetric variation [16].

Given the uncertainty in target delineation within collapsed lung, as well as limitations in tumor visualization across individual respiratory phases on 4D-CT, a generous isotropic PTV margin of 1 cm was applied to account for geometric uncertainties, including intrafraction ITV motion. Typically, when using a motion-encompassing approach with a clearly defined ITV from 4D-CT, a 5 mm PTV expansion is sufficient. However, in early cooperative group trials establishing lung SBRT as a standard of care, larger PTV margins of 1 cm cranio-caudally and 0.5 cm axially were used when planning on helical CT alone [4,17]. For this patient, 4D-CT simulation demonstrated limited target motion within the atelectasis, yet insufficient visualization across respiratory phases precluded accurate ITV delineation. In practical terms, although a generous PTV was applied, the volume of ventilating, functional lung tissue included within the PTV was lower than if the tumor had been located entirely within aerated lung. This strategy allowed for adequate target coverage while minimizing the risk of toxicity to normal aerated lung tissue, demonstrating a tailored approach to SBRT planning in tumors embedded within atelectasis.

Target location within atelectasis is very challenging since the tumor cannot be directly visualized at the time of treatment. The use of stable anatomic surrogates as matching structures at the time of pretreatment CBCT can greatly aid in target localization when soft-tissue contrast or respiratory motion limits direct tumor matching, ensuring reproducible setup and accurate targeting [18]. In this case, the atelectasis itself was contoured as a surrogate matching structure as well as distal segments of the right upper lobe bronchus located immediately adjacent to the tumor as confirmed by PET-CT. Matching to these surrogate structures improved geometric fidelity despite limited direct tumor visibility on CBCT, supporting safe and precise treatment delivery in this challenging clinical scenario. Utilization of SF-SBRT minimized the challenges associated with repeated setup and target localization uncertainties across multiple treatments.

## Conclusions

SF-SBRT can be safely and effectively delivered to early-stage NSCLC within atelectatic lung. PET-CT fusion facilitates accurate GTV delineation by distinguishing metabolically active tumor from surrounding atelectasis. Appropriately generous PTV margins can account for increased uncertainties in target delineation and motion while maintaining a low risk of toxicity, since the irradiated lung is non-functional. Surrogate anatomic matching structures can be used for image-guided target localization when the tumor cannot be directly visualized. This case highlights the feasibility of SF-SBRT which achieved short-term metabolic response in this challenging scenario, however long-term local control remains unclear given the patient’s death from distant progression. Further research is needed to establish standardized contouring and planning guidelines for tumors embedded within atelectasis.

## References

[1] Bartl AJ, Mahoney M, Hennon MW (2022). Systematic review of single-fraction stereotactic body radiation therapy for early stage non-small-cell lung cancer and lung oligometastases: how to stop worrying and love one and done. Cancers (Basel).

[2] Callueng JM, Baker S, Chng N (2026). Population-based outcomes of single-fraction stereotactic ablative radiotherapy for early stage non-small cell lung cancer. Clin Oncol (R Coll Radiol).

[3] Singh AK, Gomez-Suescun JA, Stephans KL (2019). One versus three fractions of stereotactic body radiation therapy for peripheral stage I to II non-small cell lung cancer: a randomized, multi-institution, phase 2 trial. Int J Radiat Oncol Biol Phys.

[4] Videtic GM, Hu C, Singh AK (2015). A randomized phase 2 study comparing 2 stereotactic body radiation therapy schedules for medically inoperable patients with stage I peripheral non-small cell lung cancer: NRG Oncology RTOG 0915 (NCCTG N0927). Int J Radiat Oncol Biol Phys.

[5] Guy CL, Weiss E, Jan N, Reshko LB, Christensen GE, Hugo GD (2016). Effect of atelectasis changes on tissue mass and dose during lung radiotherapy. Med Phys.

[6] Oken MM, Creech RH, Tormey DC (1982). Toxicity and response criteria of the Eastern Cooperative Oncology Group. Am J Clin Oncol.

[7] Mou B, Hyde D, Araujo C, Bartha L, Bergman A, Liu M (2021). Implementation of single-fraction lung stereotactic ablative radiotherapy in a multicenter provincial cancer program during the COVID-19 pandemic. Cureus.

[8] Wang N, Qiao Y, Song Y (2022). In 18F-positron emission tomography/computed tomography-guided precision radiotherapy for centrally located non-small cell lung cancer, tumor related atelectasis is a prognostic factor of survival. Front Oncol.

[9] Nestle U, Schimek-Jasch T, Kremp S (2020). Imaging-based target volume reduction in chemoradiotherapy for locally advanced non-small-cell lung cancer (PET-Plan): a multicentre, open-label, randomised, controlled trial. Lancet Oncol.

[10] Erdi YE, Rosenzweig K, Erdi AK (2002). Radiotherapy treatment planning for patients with non-small cell lung cancer using positron emission tomography (PET). Radiother Oncol.

[11] Steenbakkers RJ, Duppen JC, Fitton I (2006). Reduction of observer variation using matched CT-PET for lung cancer delineation: a three-dimensional analysis. Int J Radiat Oncol Biol Phys.

[12] Yin LJ, Yu XB, Ren YG, Gu GH, Ding TG, Lu Z (2013). Utilization of PET-CT in target volume delineation for three-dimensional conformal radiotherapy in patients with non-small cell lung cancer and atelectasis. Multidiscip Respir Med.

[13] Chirindel A, Adebahr S, Schuster D (2015). Impact of 4D-(18)FDG-PET/CT imaging on target volume delineation in SBRT patients with central versus peripheral lung tumors. Multi-reader comparative study. Radiother Oncol.

[14] (2026). Radical resection vs. ablative stereotactic radiotherapy in patients with operable stage I NSCLC (POSTILV). https://clinicaltrials.gov/study/NCT01753414.

[15] Tennyson N, Weiss E, Sleeman W, Rosu M, Jan N, Hugo GD (2017). Effect of variations in atelectasis on tumor displacement during radiation therapy for locally advanced lung cancer. Adv Radiat Oncol.

[16] Chen H, Shao Y, Gu X (2021). Geometric and dosimetric changes in tumor and lung tissue during radiotherapy for lung cancer with atelectasis. Front Oncol.

[17] Timmerman R, Paulus R, Galvin J (2010). Stereotactic body radiation therapy for inoperable early stage lung cancer. JAMA.

[18] Li HS, Kong LL, Zhang J (2012). Evaluation of the geometric accuracy of anatomic landmarks as surrogates for intrapulmonary tumors in image-guided radiotherapy. Asian Pac J Cancer Prev.

